# Elongation of very Long-Chain (>C_24_) Fatty Acids in *Clarias gariepinus*: Cloning, Functional Characterization and Tissue Expression of *elovl4* Elongases

**DOI:** 10.1007/s11745-017-4289-3

**Published:** 2017-08-30

**Authors:** Angela Oboh, Juan C. Navarro, Douglas R. Tocher, Oscar Monroig

**Affiliations:** 1 Faculty of Natural Sciences, Institute of Aquaculture, University of Stirling, Stirling, FK9 4LA Scotland, UK; 2 Department of Biological Sciences, University of Abuja, P.M.B. 117, Abuja, Nigeria; 3 Instituto de Acuicultura Torre de la Sal (IATS-CSIC), 12595 Ribera de Cabanes, Castellón Spain

**Keywords:** *Clarias gariepinus*, *elovl4*, Essential fatty acids, Biosynthesis, Very long-chain fatty acids

## Abstract

Elongation of very long-chain fatty acid 4 (Elovl4) proteins participate in the biosynthesis of very long-chain (>C_24_) saturated and polyunsaturated fatty acids (FA). Previous studies have shown that fish possess two different forms of Elovl4, termed Elovl4a and Elovl4b. The present study aimed to characterize both molecularly and functionally two *elovl4* cDNA from the African catfish *Clarias gariepinus*. The results confirmed that *C. gariepinus* possessed two *elovl4*-like elongases with high homology to two previously characterized Elovl4 from *Danio rerio*, and thus they were termed accordingly as Elovl4a and Elovl4b. The *C. gariepinus* Elovl4a and Elovl4b have open reading frames (ORF) of 945 and 915 base pairs, respectively, encoding putative proteins of 314 and 304 amino acids, respectively. Functional characterization in yeast showed both Elovl4 enzymes have activity towards all the PUFA substrates assayed (18:4n-3, 18:3n-6, 20:5n-3, 20:4n-6, 22:5n-3, 22:4n-6 and 22:6n-3), producing elongated products of up to C_36_. Moreover, the *C. gariepinus* Elovl4a and Elovl4b were able to elongate very long-chain saturated FA (VLC-SFA) as denoted by increased levels of 28:0 and longer FA in yeast transformed with *elovl4* ORF compared to control yeast. These results confirmed that *C. gariepinus* Elovl4 play important roles in the biosynthesis of very long-chain FA. Tissue distribution analysis of *elovl4* mRNAs showed both genes were widely expressed in all tissues analyzed, with high expression of *elovl4a* in pituitary and brain, whereas female gonad and pituitary had the highest expression levels for *elovl4b*.

## Introduction

Elongation of very long-chain fatty acid (Elovl) proteins catalyze the condensation reaction, regarded as the first and rate-limiting step of four sequential reactions required for the elongation of fatty acids (FA) [1, 2]. Seven members (Elovl 1–7) with similar motifs make up the Elovl protein family in vertebrates, although only Elovl2, Elovl4 and Elovl5 have been proven to have polyunsaturated fatty acids (PUFA) as substrates for elongation [1, 2]. Importantly, the complement of Elovl, along with that of fatty acyl desaturases (Fads), determines the ability of species to biosynthesize physiologically essential fatty acids (EFA) such as eicosapentaenoic acid (EPA, 20:5n-3), arachidonic acid (ARA, 20:4n-6) and docosahexaenoic acid (DHA, 22:6n-3) [3]. Fish have arguably been the group of organisms in which the most comprehensive characterization of Elovl gene repertoire and function has been conducted, particularly farmed species [4]. These studies have shown that Elovl5 elongates predominantly C_18_ and C_20_ PUFA, whilst Elovl2 preferentially elongates C_20_ and C_22_ PUFA [4], thus denoting somewhat overlapping functionalities that are likely to derive from a common evolutionary origin [5]. However, the substrate specificities of Elovl4 proteins from vertebrates including fish have remained more elusive [4].

Cloning and functional characterization of a teleost Elovl4 was first carried out in zebrafish *Danio rerio* [6]. It was shown that two Elovl4 genes, termed Elovl4a and Elovl4b, were present, in contrast to mammals in which only a single Elovl4 had been reported [7]. Interestingly, both *D. rerio* Elovl4 showed an ability to elongate saturated fatty acids, but only Elovl4b appeared to have a role in the biosynthesis of very long-chain (>C_24_) polyunsaturated fatty acids (VLC-PUFA) [6]. Since this pioneer study in fish, further *elovl4* cDNA sequences have been studied in a variety of species including Atlantic salmon, Nibe croaker, orange-spotted grouper and rabbitfish [8–11]. Interestingly, with the exception of the zebrafish *elovl4a* [6], all *elovl4* cDNA cloned from other teleost fish species have been confirmed to be orthologues of the zebrafish *elovl4b*, although *in silico* searches indicated that virtually all teleosts possess at least one copy of both *elovl4a* and *elovl4b* [4]. Recently, two further elongases termed *elovl4c*-*1* and *elovl4c*-*2* were identified from the Atlantic cod, *Gadus morhua*, although their functionalities remain to be elucidated [12]. In addition to the differences in substrate specificities, further evidence suggesting that Elovl4a and Elovl4b participate in different biological processes was provided by tissue expression patterns suggesting *elovl4a* was highly expressed in the brain, whereas *elovl4b* was highly expressed in eye (retina) and gonads [6, 12]. These results were consistent with studies on mammals indicating that these tissues are important sites for very long-chain fatty acid biosynthesis. Thus, very long-chain (>C_24_) saturated fatty acids (VLC-SFA) have been shown to play key roles in skin permeability barrier formation and thus essential for neonatal survival [13–15], whereas VLC-PUFA are essential in phototransduction and male fertility [16–18].

An interesting trait that apparently differentiates fish Elovl4 from non-fish vertebrate Elovl4 orthologues is the ability of the former to catalyze the elongation of C_22_ PUFA substrates to C_24_ products. In particular, all fish Elovl4b characterized to date have shown the ability to efficiently elongate 22:5n-3 to 24:5n-3, a critical enzymatic step in the biosynthesis of DHA through the Sprecher pathway [19]. The acquisition or retention of such an ability by some fish Elovl4 has been hypothesized to compensate the loss of *elovl2* during the evolution history of some teleost lineages encompassing the vast majority of farmed marine fish species [6, 10, 11, 20]. Indeed, the apparent absence of *elovl2*, along with that of key desaturation activities, has been regarded as molecular evidence accounting for the low capacity of marine fish species to biosynthesize EPA, ARA and DHA [21].

Our overall aim is to elucidate the repertoire and function of genes encoding *elovl* and *fads* enzymes involved in the biosynthesis of essential fatty acids in the African catfish, *Clarias gariepinus*, a commercially important species in Sub-Saharan African aquaculture [22]. *C. gariepinus* are freshwater fish with a variety of characteristics that makes them ideal for fish farming. African catfish *C. gariepinus* is a fast growing species, can be cultured at high densities and tolerates poor water quality due to the possession of accessory air-breathing organs [23, 24]. *C. gariepinus* is an omnivorous fish and, while in the wild they feed on insects, crustaceans, worms, gastropods, fishes and plants, they accept a wide range of feed ingredients in captivity [24]. With regards to PUFA biosynthesizing enzymes, Agaba *et al*. [25] characterized an Elovl5 from *C. gariepinus* that was primarily active towards C_18–20_ PUFA substrates. More recently, we successfully isolated and functionally characterized an Elovl2 elongase with preference towards C_20–22_ PUFA substrates, as well as a Fads2 desaturase with dual Δ6Δ5 activity [26]. In the present study, we characterized, both molecularly and functionally, two *elovl4* cDNA from *C. gariepinus* and investigated their tissue expression patterns.

## Materials and Methods

### Sample Collection and RNA Preparation

Tissue samples used in this study were obtained from adult *C. gariepinus* specimens (~1.8 kg) raised in the tropical aquarium of the Institute of Aquaculture, University of Stirling, UK, and fed on standard salmonid diets. All experimental procedures were approved by the Animal Welfare and Ethical Review Board of the University of Stirling and conducted in compliance with the Animals Scientific Procedures Act 1986 (Home Office Code of Practice. HMSO: London January 1997). Eight *C. gariepinus* individuals (four male and four female) were sacrificed with an overdose of tricaine methanesulfonate (MS222) before collection of tissue samples including liver, eye, intestine, pituitary, testis, ovary, skin, muscle, gills, kidney, head kidney, accessory breathing organ (ABO), adipose tissue, stomach, heart and brain. The samples were immediately preserved in RNA stabilization buffer (3.6 M ammonium sulphate, 18 mM sodium citrate, 15 mM EDTA, pH 5.2) and stored at −80 °C until required. Total RNA was extracted from tissues derived from individual fish using TRI Reagent^®^ (Sigma-Aldrich, USA). Purity and concentration of total RNA was assessed using the NanoDrop^®^ (Labtech International ND-1000 spectrophotometer) and integrity was assessed on an agarose gel. First strand complementary DNA (cDNA) was synthesized from 1 µg total RNA using High Capacity cDNA Reverse Transcription Kit (Applied Biosystems™, USA) following the manufacturer’s instructions.

### Molecular Cloning of elovl4 cDNA

Amplification of partial fragments of the genes was achieved by polymerase chain reaction (PCR) using a mixture of cDNA from eye and brain as template. For amplification of the first fragment of the *C. gariepinus elovl4a*, the primers UniE4aF (5ʹ-CTCTTCCTCTGGCTGGGG-3ʹ) and UniE4aR (5ʹ-TATGTCTGGTAGTAGAAGTTCC-3ʹ) were designed on conserved regions after alignment (BioEdit v7.0.9, Tom Hall, Department of Microbiology, North Carolina State University, USA) of *elovl4a*-like sequences from *D. rerio* (gb|NM_200796.1|), *G. morhua* (KF964008.1), *Takifugu rubripes* (gb|XM_003965960.1|) and *Ictalurus punctatus* (gb|JT417431.1|). Similarly, *elovl4b* homologous sequences from *Siganus canaliculatus* (gb|JF320823.1|), *Rachycentron canadum* (gb|HM026361.1|), *Salmo salar* (gb|NM_001195552.1|) and *I. punctatus* (gb|JT405661.1|) were aligned to design primers UniE4bF (5ʹ-TAGCAGACAAGCGGGTGG-3ʹ) and UniE4bR (5ʹ-CAAAGAGGATGATGAAGGTGA-3ʹ) used for the amplification of the first fragment of *C. gariepinus elovl4b*. PCR conditions consisted of an initial denaturation step at 95 °C for 2 min, followed by 35 cycles of denaturation at 95 °C for 30 s, annealing at 55 °C for 30 s, extension at 72 °C for 55 s, followed by a final extension at 72 °C for 7 min. PCR fragments were purified using the Illustra GFX PCR DNA/gel band purification kit (GE Healthcare, UK), and sequenced at GATC Biotech Ltd. (Germany).

Gene-specific primers were designed to obtain full-length cDNA by 5ʹ and 3ʹ Rapid Amplification of cDNA Ends (RACE) PCR (FirstChoice^®^ RLM-RACE RNA ligase mediated RACE kit, Ambion^®^, Life Technologies™, USA). Positive RACE PCR products were identified by sequencing (GATC Biotech Ltd). The full nucleotide sequences of both *elovl4* cDNA sequences were obtained by aligning sequences of the first fragments, together with those of the 5ʹ and 3ʹ RACE PCR positive products (BioEdit). All primers used in RACE PCR are listed in Table 1.

**Table 1:** Sequences of primers used for molecular cloning of full-length cDNA and tissue expression analysis (qPCR) of *Clarias gariepinus elovl4a* and *elovl4b*

Name	Direction	Sequence
*Initial cDNA cloning*		
UniE4aF	Forward	5ʹ-CTCTTCCTCTGGCTGGGG-3ʹ
UniE4aR	Reverse	5ʹ-TATGTCTGGTAGTAGAAGTTCC-3ʹ
UniE4bF	Forward	5ʹ-TAGCAGACAAGCGGGTGG-3ʹ
UniE4bR	Reverse	5ʹ-CAAAGAGGATGATGAAGGTGA-3ʹ
*5*ʹ *RACE*		
CGRE4aR3	Reverse	5ʹ-GCAAGGAAGAGCTCTTTGAAG-3ʹ
CGRE4aR2	Reverse	5ʹ-ACAATTAGGGTCTTCCTGAGCT-3ʹ
CGRE4bR3	Reverse	5ʹ-GCAGCACCATGCTGAAGT-3ʹ
CGRE4bR2	Reverse	5ʹ-TGAAAGCGTCTCGGTGCT-3ʹ
*3*ʹ *RACE*		
CGRE4aF1	Forward	5ʹ-TCATTGTCCTCTTTGGGAACT-3ʹ
CGRE4aF2	Forward	5ʹ-GCACTGGTGTCTGATTGGTTAT-3ʹ
CGRE4bF2	Forward	5ʹ-CTCACTCGCTGTACTCCGG-3ʹ
CGRE4bF3	Forward	5ʹ-CCAGTTCCATGTCACAATCG-3ʹ
*ORF cloning*		
CGE4aVF	Forward	5ʹ-CCC*GGATCC*AAGATGGATATTGTAACAC-3ʹ
CGE4aVR	Reverse	5ʹ-CCG*CTCGAG*CTAGTCCCGCTTTGCCCTGCC-3ʹ
CGE4bVF	Forward	5ʹ-CCC*GGATCC*AACATGGAAACGGTGCTTC-3ʹ
CGE4bVR	Reverse	5ʹ-CCG*CTCGAG*TCACTCCCTCTTTGTTCGTTCC-3ʹ
*qPCR*		
CGqE4aF1	Forward	5ʹ-GAGATGCAGAAGCAGGCATA-3ʹ
CGqE4aR1	Reverse	5ʹ-TTGAGCCTCCTCCAAACAGT-3ʹ
CGqE4bF1	Forward	5ʹ-GAGGAACGCACTGGGAACT-3ʹ
CGqE4bR1	Reverse	5ʹ-AAACGCCATCTATCCCATTG-3ʹ
28SrRNAF1	Forward	5ʹ-GTCCTTCTGATGGAGGCTCA-3ʹ
28SrRNAR1	Reverse	5ʹ-CGTGCCGGTATTTAGCCTTA-3ʹ

Restriction sites for *Bam*HI (forward) and *Xho*I (reverse) are italicized

### Sequence and Phylogenetic Analysis

The deduced amino acid (aa) sequences of both *C. gariepinus elovl4* cDNA sequences were compared to corresponding orthologues from other vertebrate species by calculating the identity scores using the EMBOSS Needle Pairwise Sequence Alignment tool (http://www.ebi.ac.uk/Tools/psa/emboss_needle/). Phylogenetic analysis of the deduced aa sequences of the Elovl4 proteins from *C. gariepinus* and Elovl from a variety of vertebrate species was performed by constructing a tree using the neighbor-joining method [27] with MEGA 6.0 software (www.megasoftware.net). Confidence in the resulting tree branch topology was measured by bootstrapping through 1000 iterations.

### Functional Characterization of *C*. *gariepinus Elovl4a* and *Elovl4b* by Heterologous Expression in Saccharomyces cerevisiae

PCR fragments corresponding to the open reading frame (ORF) of *C. gariepinus* newly cloned *elovl4* cDNA were amplified from a mixture of cDNA synthesized from eye and brain RNA, using the high fidelity *Pfu* DNA polymerase (Promega, USA) with primers containing *Bam*HI (forward) and *Xho*I (reverse) restriction sites (Table 1). PCR conditions consisted of an initial denaturation at 95 °C for 2 min, followed by 32 cycles of denaturation at 95 °C for 30 s, annealing at 55 °C for 30 s, extension at 72 °C for 2 min followed by a final extension at 72 °C for 7 min. The DNA fragments obtained were purified as described above, digested with the appropriate restriction enzymes (New England Biolabs, UK), and ligated into the similarly digested pYES2 expression vector (Invitrogen, UK) to produce the plasmid constructs pYES2-*elovl4a* and pYES2-*elovl4b*.

Yeast competent cells InvSc1 (Invitrogen) were transformed with pYES2-*elovl4a* and pYES2-*elovl4b* using the S.c. EasyComp™ Transformation Kit (Invitrogen). Selection of yeast containing the pYES2 constructs was done on *S. cerevisiae* minimal medium minus uracil (SCMM^-ura^) plates. One single yeast colony was grown in SCMM^-ura^ broth for 2 days at 30 °C, and subsequently subcultured in individual Erlenmeyer flasks until optical density measured at a wavelength of 600 nm (OD_600_) reached 1, after which galactose (2%, w/v) and a PUFA substrate at a final concentration of 0.50 mM (C_18_), 0.75 mM (C_20_) and 1.0 mM (C_22_) were added. The FA substrates included stearidonic acid (18:4n-3), gamma-linolenic acid (18:3n-6), EPA (20:5n-3), ARA (20:4n-6), docosapentaenoic acid (22:5n-3), docosatetraenoic acid (22:4n-6) and DHA (22:6n-3). In addition to exogenously added PUFA substrates, some Elovl4 have been shown to elongate saturated FA [6]. Consequently, the ability of *C. gariepinus* Elovl4 enzymes to elongate yeast endogenous saturated FA was investigated. For that purpose, the saturated FA profiles of yeast transformed with empty pYES2 vector and those of yeast transformed with either pYES2-*elovl4a* or pYES2-*elovl4b* were compared after growing the yeast without addition of any substrate. After 2 days, yeast were harvested, washed twice with doubled distilled water and freeze-dried until further analysis. All FA substrates (>98–99% pure) used for the functional characterization assays, except for stearidonic acid (18:4n-3) were obtained from Nu-Chek Prep, Inc (Elysian, MN, USA). Stearidonic acid (>99% pure) and yeast culture reagents including galactose, nitrogen base, raffinose, tergitol NP-40 and uracil dropout medium were obtained from Sigma-Aldrich (Poole, UK).

### Fatty Acid Analysis of Yeast

Total lipids extracted from freeze-dried samples of yeast [28] were used to prepare fatty acid methyl esters (FAME) as described in detailed previously [26]. Identification of the peaks was carried out as described by Li *et al*. [29]. Briefly, FAME were identified and quantified after splitless injection and run in temperature programming, in an Agilent 6850 gas chromatograph system, equipped with a Sapiens-5MS (30 m × 0.25 µm × 0.25 µm) capillary column (Teknokroma, Spain) coupled to a 5975 series mass spectrometer detector (Agilent Technologies, USA). The elongation of endogenous saturated FA was assessed by comparison of the areas of the FA of control yeast with those of yeast transformed with either pYES2-*elovl4a* or pYES2-*elovl4b*. As described in detail by Li *et al*. [29], the elongation conversions of exogenously added PUFA substrates (18:4n-3, 18:3n-6, 18:4n-3, 20:5n-3, 20:4n-6, 22:5n-3 and 22:6n-3) were calculated by the step-wise proportion of substrate FA converted to elongated product as [areas of first product and longer chain products/(areas of all products with longer chain than substrate + substrate area)] × 100.

### Gene Expression Analysis

Expression of the newly cloned *C. gariepinus elovl4* cDNAs was measured by quantitative real-time PCR (qPCR). RNA extraction from *C. gariepinus* tissues (four male and four females) and cDNA synthesis were carried out as described above. PCR amplicons of each gene cloned into PCR 2.1 vector (TA cloning^®^ kit, Invitrogen, Life Technologies™, USA) were linearized, quantified and serial-diluted to generate a standard curve of known copy numbers for quantification [26]. All qPCR amplifications were carried out in duplicate using Biometra Thermocycler (Analytik Jena Company, Germany) and Luminaris Color Higreen qPCR master mix (Thermo Scientific, USA) following the manufacturer’s instructions. QPCR were performed in a final volume of 20 μl containing 5 μl diluted (1/20) cDNA, 1 μl (10 μM) of each primer, 3 μl nuclease free water and 10 μl Luminaris Color Higreen qPCR master mix. The qPCR conditions were 50 °C for 2 min, 95 °C for 10 min followed by 35 cycles of denaturation step at 95 °C for 15 s, annealing at 59 °C for 30 s and extension at 72 °C for 30 s. After amplification, a dissociation curve of 0.5 °C increments from 60 to 90 °C was performed, enabling confirmation of a single product in each reaction. Identity of the qPCR products was further confirmed by DNA sequencing of selected samples (GATC Biotech Ltd.). Negative controls (no template control, NTC) containing no cDNA were systematically run. Absolute copy number of the target and reference gene in each sample was calculated from the linear standard curve constructed. Normalization of each target gene was carried out by dividing the absolute copy number of the candidate gene by the absolute copy number of the reference gene, 28S rRNA (gb|AF323692.1|). In order to prepare solutions of known copy numbers, DNA concentration of linearized PCR 2.1 vectors containing a fragment of either candidate or reference genes was determined, and their molecular weights estimated as 660 g × length in base pair (bp) of the plasmid constructs. Primers used for qPCR analysis are presented in Table 1.

### Statistical Analysis

Tissue expression (qPCR) results were expressed as mean normalized ratios (*n* = 4) (±SE) corresponding to the ratio between the copy numbers of the target genes (*elovl4a* and *elovl4b*) and the copy numbers of the reference gene, 28S rRNA. Differences in gene expression among tissues were analyzed by one-way analysis of variance (ANOVA) followed by Tukey’s HSD test at a significance level of *P* ≤ 0.05 (IBM SPSS Statistics 21, USA).

## Results

### Elovl4 Sequence and Phylogenetic Analysis

The sequence and phylogenetic analysis revealed that *C. gariepinus* possesses two *elovl4* cDNAs with homology to the *D. rerio* Elovl4 proteins [6] and, for consistency, were termed as *elovl4a* and *elovl4b*. The full-length of the *C. gariepinus elovl4a* cDNA consisted of 1403 bp that contained an ORF of 945 bp encoding a putative protein of 314 aa. Whereas, the full-length of the *C. gariepinus elovl4b* was 1181 bp, with a 915 bp ORF encoding a putative protein of 304 aa. Both cDNA sequences have been deposited with the GenBank database under the accession number KY801284 (*elovl4a*) and KY801285 (*elovl4b*).

**Fig. 1:**
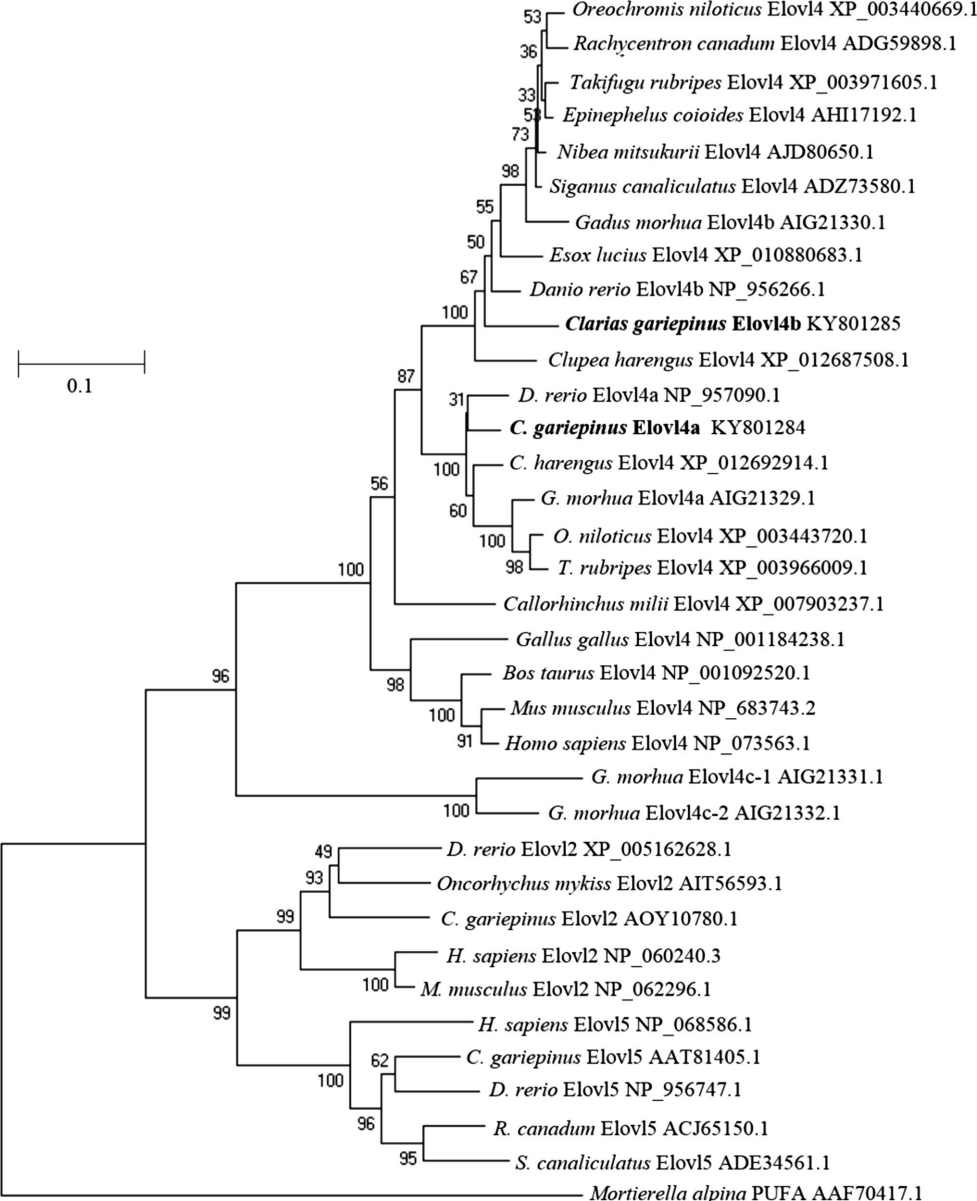
Phylogenetic tree comparing the deduced amino acid sequences of *Clarias gariepinus* Elovl4a and Elovl4b (highlighted in *bold*) with Elovl4, Elovl2 and Elovl5 sequences from a range of vertebrates. The tree was constructed using the neighbor-joining method [27] with the MEGA 6.0 software. The *numbers* represent the frequencies (%) with which the tree topology presented was replicated after 1000 iterations. The *Mortierella alpina* PUFA elongase was included in the analysis as outgroup sequence to construct the rooted tree

Phylogenetic analysis showed that *C. gariepinus* Elovl4 proteins grouped together with orthologues from a variety of vertebrates, with Elovl2 and Elovl5 sequences clustering separately (Fig. 1). Within the teleost Elovl4, two distinct clusters containing each of the two Elovl4 from *C. gariepinus* could be identified. In one cluster, the *C. gariepinus* Elovl4a grouped closely with Elovl4a-like sequences from *D. rerio* (gb|NP_957090.1|), *G. morhua* (gb|AIG21330.1|), *C. harengus* (gb|XP_012692914.1|), *Oreochromis niloticus* (gb|XP_003443720.1|) and *T. rubripes* (gb|XP_003966009.1|). In the other, the *C. gariepinus* Elovl4b grouped with Elovl4b-like sequences from *D. rerio* (gb|NP_956266.1|), *N. mitsukuri* (gb|AJD80650.1|), *R. canadum* (ADG59898.1) and *G. morhua* (gb|AIG213329.1|). These results confirmed that the newly cloned *elovl* cDNA from *C. gariepinus* encoded Elovl4a and Elovl4b proteins. Interestingly, the two so-called “Elovl4c” previously reported in *G. morhua* [12] grouped separately from all vertebrate Elovl4 (Fig. 1).

**Fig. 2:**
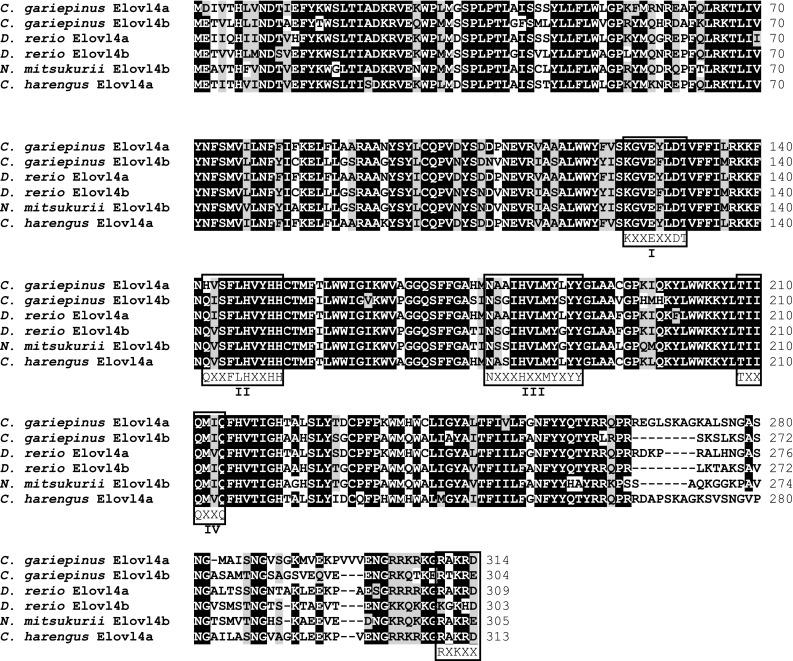
ClustalW amino acid alignment of the deduced *Clarias gariepinus* Elovl4 proteins with orthologues from *Danio rerio* (Elovl4a, gb|NP_957090.1|; Elovl4b, gb |NP_956266.1|), *Nibea mitsukurii* (gb|AJD80650.1|) and *Clupea harengus* (gb|XP_012692914.1|). Identical residues are *shaded black* and similar residues (based on the Blosum62 matrix, using ClustalW default parameters) are *shaded grey*. Indicated are four (i–iv) conserved motif of elongases: (i) KXXEXXDT, (ii) QXXFLHXXHH, (iii) NXXXHXXMYXYY and (iv) TXXQXXQ. The putative endoplasmic reticulum (ER) retrieval signal RXKXX at C-terminus is also indicated [25]

Sequence analysis of the *C. gariepinus* putative Elovl4a and Elovl4b proteins showed that both possessed all the characteristic features of Elovl family members including a single histidine dideoxy binding motif HXXHH, the putative endoplasmic reticulum (ER) retrieval signal with an arginine (R) and lysine (K) residue at the carboxyl terminus, RXKXX) and multiple regions containing similar motifs such as (1) KXXEXXDT, (2) QXXFLHXXHH, (3) NXXXHXXMYXYY, (4) TXXQXXQ (Fig. 2) [2, 25]. The deduced aa sequences from the *C. gariepinus* Elovl4a and Elovl4b were 70.7% similar to each other. Comparing the aa sequences of *C. gariepinus* Elovl4 deduced proteins with other fish Elovl4 sequences revealed that Elovl4a shared highest identities with *Clupea harengus* (gb|XP_012692914.1|) (87.0%) and *D. rerio* Elovl4a (85.7%), whereas *C. gariepinus* Elovl4b shared highest identities with *D. rerio* Elovl4b (83.6%) and *Nibea mitsukuri* Elovl4 (gb|AJD80650.1|) (81.0%). The aa sequence of *C. gariepinus* Elovl4a shared 41.5% and 38.0%, respectively, with previously described *C. gariepinus* Elovl5 and Elovl2 elongases, while identity scores of 43 and 40.8%, respectively, were obtained for Elovl4b.

### Functional Characterization of *C*. *gariepinus Elovl4* in Yeast

The role of the *C. gariepinus* Elovl4 enzymes in the elongation of very long-chain saturated FA was assessed by comparison of the saturated (≥C_24_) FA profiles of control yeast transformed with empty pYES2 with those of yeast transformed with either pYES2-*elovl4a* or pYES2-*elovl4b* and grown in all cases in the absence of exogenously added FA. The results confirmed that the *C. gariepinus* Elovl4 enzymes were involved in the biosynthesis of very long-chain saturated FA since yeast expressing both *elovl4a* and *elovl4b* generally contained higher levels of saturated FA ≥ C_28_. More specifically, yeast expressing the *C. gariepinus elovl4a* had significantly higher levels of 28:0, 30:0 and 32:0 compared to control yeast, whereas yeast expressing the *C. gariepinus elovl4b* contained higher levels of 28:0 and 32:0 compared to controls (Table 2).

**Table 2:** Functional characterization of *Clarias gariepinus* Elovl4 elongases: role in biosynthesis of very long-chain saturated fatty acids (FA)

FA	Control	Elovl4a	Elovl4b
24:0	1.19 ± 0.10^a^	1.60 ± 0.25^b^	1.51 ± 0.08^b^
26:0	23.46 ± 1.15^a^	22.49 ± 0.76^a^	26.82 ± 4.81^a^
28:0	0.95 ± 0.19^a^	4.42 ± 0.62^b^	2.23 ± 0.33^b^
30:0	0.23 ± 0.06^a^	2.51 ± 0.44^b^	0.48 ± 0.05^a^
32:0	0.04 ± 0.01^a^	0.40 ± 0.02^b^	0.11 ± 0.04^b^

Results are expressed as an area percentage of total saturated FA ≥ C_24_ found in yeast transformed with either *C. gariepinus elovl4* coding regions or empty pYES2 vector (control)

**Fig. 3:**
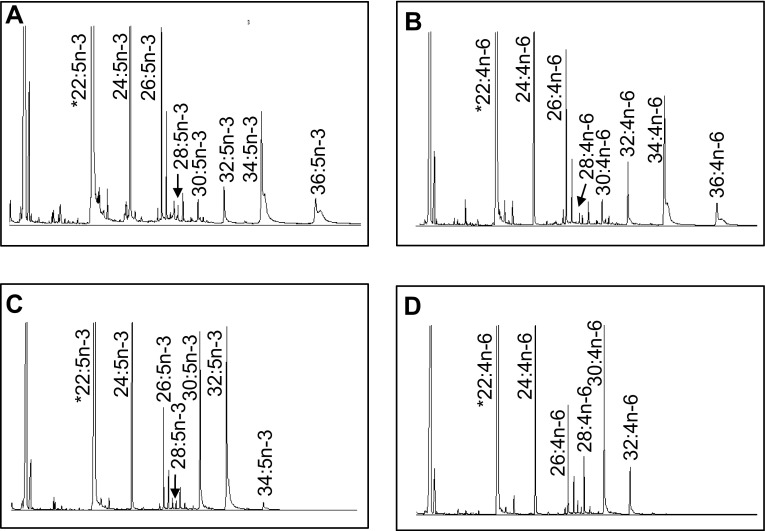
Functional characterization of the newly cloned *Clarias gariepinus* Elovl4a (**a**, **b**) and Elovl4b (**c**, **d**) in yeast (*Saccharomyces cerevisiae*). The fatty acid profiles of yeast transformed with pYES2 containing the coding sequence of *elovl4a* and *elovl4b* were determined after the yeast were grown in the presence of one of the exogenously added substrates 22:5n-3 (**a**, **c**), and 22:4n-6 (**b**, **d**). The first peak (with *asterisk*) is derived from the exogenously added substrates. The elongation products are indicated accordingly in *each panel*

The role of the *C. gariepinus* Elovl4 enzymes in VLC-PUFA biosynthesis was investigated by growing transgenic yeast expressing the *C. gariepinus elovl4a* and *elovl4b* cDNA in the presence of potential PUFA substrates (Fig. 3). While transgenic yeast were able to elongate exogenously added PUFA substrates with chain lengths ranging from C_18_ to C_22_, the conversions were markedly higher for longer chain substrates (Table 3). For Elovl4a, step-wise elongation products derived from exogenously supplemented PUFA and with C_28−34_ were very efficiently elongated as denoted by high conversions that were often above 80% (Table 3). In contrast, the *C. gariepinus* Elovl4b was generally less active in the yeast expression system, leading to elongation products with a maximum length of C_34_ and with generally lower conversions compared to Elovl4a (Table 3). As an exception, the Elovl4b was very efficient at utilizing 22:6n-3 to produce intermediate elongation products up to 32:6n-3. It is important to note that both Elovl4 enzymes were able to produce 24:5n-3 from 22:5n-3 supplied directly or converted from exogenously supplied 20:5n-3 (Table 3; Fig. 3).

**Table 3:** Functional characterization of *Clarias gariepinus* Elovl4 elongases: role in biosynthesis of very long-chain polyunsaturated fatty acids (VLC-PUFA)

FA substrate	Product	% Conversion		Elongation
		Elovl4a	Elovl4b	
18:4n-3	20:4n-3	3.7	2.0	C18 → 36
	22:4n-3	26.8	6.4	C20 → 36
	24:4n-3	53.1	7.9	C22 → 36
	26:4n-3	62.9	6.8	C24 → 36
	28:4n-3	100	3.7	C26 → 36
	30:4n-3	100	48.9	C28 → 36
	32:4n-3	91.2	48.4	C30 → 36
	34:4n-3	83.6	1.4	C32 → 36
	36:4n-3	7.7	N.D.	C34 → 36
18:3n-6	20:3n-6	6.0	3.0	C18 → 36
	22:3n-6	49.5	9.9	C20 → 36
	24:3n-6	73.2	12.2	C22 → 36
	26:3n-6	80.2	29.4	C24 → 36
	28:3n-6	100	100	C26 → 36
	30:3n-6	100	100	C28 → 36
	32:3n-6	100	51.7	C30 → 36
	34:3n-6	69.5	N.D.	C32 → 36
	36:3n-6	8.1	N.D.	C34 → 36
20:5n-3	22:5n-3	20.4	6.3	C20 → 36
	24:5n-3	41.4	13.2	C22 → 36
	26:5n-3	55.8	4.7	C24 → 36
	28:5n-3	100	100	C26 → 36
	30:5n-3	100	19.3	C28 → 36
	32:5n-3	83.3	69.9	C30 → 36
	34:5n-3	93.4	12.0	C32 → 36
	36:5n-3	48.1	N.D.	C34 → 36
20:4n-6	22:4n-6	26.0	7.5	C20 → 36
	24:4n-6	54.6	15.9	C22 → 36
	26:4n-6	70.4	10.9	C24 → 36
	28:4n-6	85.4	9.9	C26 → 36
	30:4n-6	99.2	63.1	C28 → 36
	32:4n-6	96.5	28.4	C30 → 36
	34:4n-6	87.1	N.D.	C32 → 36
	36:4n-6	27.4	N.D.	C34 → 36
22:5n-3	24:5n-3	14.2	5.2	C22 → 36
	26:5n-3	51.1	3.9	C24 → 36
	28:5n-3	83.4	2.4	C26 → 36
	30:5n-3	98.3	31.0	C28 → 36
	32:5n-3	96.5	64.4	C30 → 36
	34:5n-3	89.8	5.6	C32 → 36
	36:5n-3	34.3	N.D.	C34 → 36
22:4n-6	24:4n-6	19.1	7.6	C22 → 36
	26:4n-6	69.9	9.9	C24 → 36
	28:4n-6	87.0	9.3	C26 → 36
	30:4n-6	99.2	67.7	C28 → 36
	32:4n-6	96.1	24.0	C30 → 36
	34:4n-6	83.8	N.D.	C32 → 36
	36:4n-6	24.3	N.D.	C34 → 36
22:6n-3	24:6n-3	0.8	0.9	C22 → 32
	26:6n-3	N.D.	100	C24 → 32
	28:6n-3	N.D.	100	C26 → 32
	30:6n-3	N.D.	100	C28 → 32
	32:6n-3	N.D.	43.7	C30 → 32

*Saccharomyces cerevisiae* transformed with empty pYES2 vector (control) or pYES2 vector containing *C. gariepinus elovl4* coding region were grown in the presence of one exogenously added polyunsaturated fatty acid (PUFA) substrate C_18_ (18:4n-3 and 18:3n-6), C_20_ (20:5n-3 and 20:4n-6) and C_22_ (22:5n-3, 22:4n-6 and 22:6n-3). Conversions were calculated for each stepwise elongation according to the formula [areas of first products and longer chain products/(areas of all products with longer chain than substrate + substrate area)] × 100. The substrate FA varies as indicated in each step-wise elongation

*ND* not detected

### Tissue Expression Analysis of C. gariepinus elovl4a and elovl4b

**Fig. 4:**
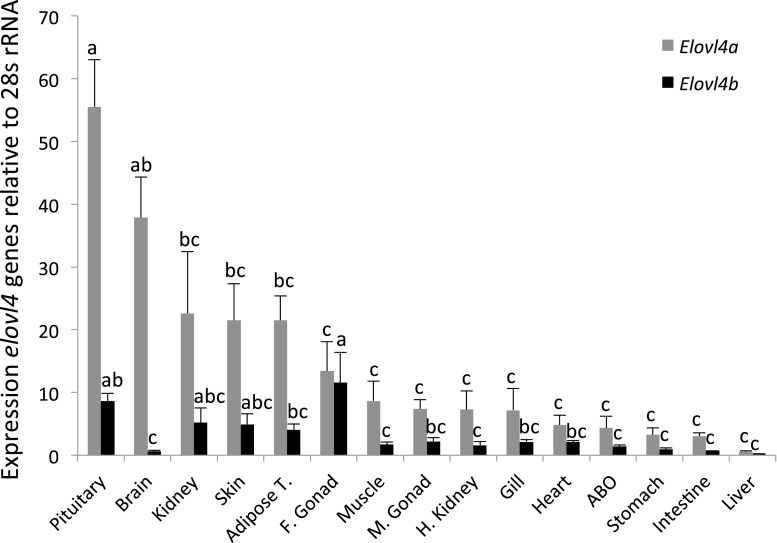
Tissue distribution of *Clarias gariepinus elovl4a* and *elovl4b* transcripts. Expression levels quantified for each target gene were normalized with the expression of the reference gene 28 s rRNA. Data are reported as mean values with their standard errors (*n* = 4). Within each target gene, *different letters* indicate statistically significant differences in expression level among tissues (ANOVA and Tukey’s HSD post hoc tests). *ABO* accessory breathing organ, *Adipose T*. adipose tissue, *F. Gonad* female gonad, *M. Gonad* male gonad

Tissue distribution analysis of *elovl4* mRNA measured by qPCR indicated both genes were expressed in all tissues analyzed, with high expression of *elovl4a* detected in pituitary and brain, whereas *elovl4b* expression was highest in female gonad and pituitary (Fig. 4). Lowest expression signals for both *elovl4* were recorded in liver.

## Discussion

Elovl enzymes with a role in long-chain (≥C_20_) PUFA (LC-PUFA) biosynthesis have been investigated extensively in fish [4], particularly in farmed species in which current diet formulations including vegetable oils might compromise the provision of EFA [30]. While Elovl5 and Elovl2 have been regarded as key elongases within LC-PUFA biosynthetic pathways, Elovl4 has received less attention despite the key role that these elongases has in crucial physiological processes including vision, reproduction and neuronal functions of vertebrates [7, 31, 32]. The present study confirmed that the *C. gariepinus* Elovl4 enzymes play critical roles in the biosynthesis of very long-chain saturated fatty acids (VLC-SFA) and PUFA (VLC-PUFA), and may also participate in the biosynthesis of DHA from EPA.

Phylogenetic analysis confirmed two isoforms of Elovl4 (Elovl4a and Elovl4b) were isolated. The Elovl4 proteins, although similar, were separated into different branches of the phylogenetic tree. The Elovl4a protein formed a group with *D. rerio* Elovl4a and other Elovl4 separate from the group consisting of *C. gariepinus* Elovl4b and Elovl4b from fish species including *D. rerio*. This is in agreement with *in silico* studies that suggested all teleosts possess both types of Elovl4 [4]. The functionally uncharacterized putative Elovl4c reported in *G. morhua* formed a group separate from all other Elovl4 sequences and therefore it is uncertain if these are true Elovl4 proteins. Functional characterization of these genes is required to confirm this.

Different functions were determined for the *C. gariepinus* Elovl4 isoforms. It was confirmed that the *C. gariepinus* Elovl4a and Elovl4b participate in the biosynthesis of VLC-SFA. Thus, yeast expressing both *elovl4a* and *elovl4b* had increased levels of VLC-SFA with C_28–32_ compared to control yeast. These results were consistent with elongation abilities of some teleost Elovl4 reported previously although, in some species like *S. salar* and *R. canadum*, Elovl4 were able to elongate up to 36:0 [6, 8, 33]. VLC-SFA play important roles in skin permeability of mammals [13, 15] and are incorporated into sphingolipids in the brain, although their role in the brain is yet to be ascertained [31]. In fish, the physiological functions of VLC-SFA have been barely investigated, although it is reasonable to believe that these compounds also have important roles in brain function of teleosts. This is supported by the high expression signal of both *elovl4* isoforms in the head region of zebrafish embryos [6], and the high expression levels in brain of certain *elovl4* with the ability to biosynthesize VLC-SFA like Elovl4a from zebrafish [6] and the herein characterized *C. gariepinus*. The existence of neurons within the hypophysis, specifically in the posterior part (neurohypophysis), may likely explain the high expression of *elovl4a* and *elovl4b* observed in the present study. Other *C. gariepinus* tissues analyzed also contained transcripts of *elovl4a*, indicating a widespread distribution as previously reported for the zebrafish *D. rerio elovl4a*, the only *elovl4a*-like sequence so far characterized in teleosts [6]. With regards to *elovl4b*, transcripts were also detected in all tissues analyzed, thus confirming a widespread distribution as described in cobia [33] and Atlantic salmon [8]. In contrast, relatively restricted tissue distributions of *elovl4b* were described in zebrafish [6] and rabbitfish [11], species in which photoreception tissues such as eye (retina) and pineal gland appear to be the major sites of Elovl4b activity [6, 11]. Unfortunately, we could not analyze the expression of the target genes in eye or pineal, although preliminary experiments indicated that both *C. gariepinus elovl4* were expressed in the eye. Furthermore, the *C. gariepinus elovl4b* was highly expressed in female gonads suggesting that, in addition to the role it may play in male reproduction of mammals, as VLC-PUFA determines male fertility [18, 31], fish Elovl4b might also have important functions in female reproduction. The above described expression patterns of the *C. gariepinus elovl4* genes, together with those tissues containing marked amounts of VLC-PUFA [31, 34], are in agreement with the roles that both Elovl4 play in the biosynthesis of VLC-PUFA in *C. gariepinus*.

Both Elovl4 showed the ability to biosynthesize VLC-PUFA of up to 34–36 carbons through consecutive elongations from all PUFA assayed including compounds with different chain lengths (C_18–22_) and series (n-3 and n-6). While this is a common trait among Elovl4b-like enzymes [6, 8, 11, 29, 33], the ability of the *C. gariepinus* Elovl4a to produce VLC-PUFA up to 36 carbons was somewhat unexpected since the only Elovl4a characterized so far from *D. rerio* showed little ability to biosynthesize VLC-PUFA [6]. Whereas current evidence does not allow us to clarify which of the two Elovl4a phenotypes (*D. rerio* or *C. gariepinus*) is more prevalent among teleosts, the apparent differences might be in response to ecological and evolutionary factors as previously hypothesized for both elongases [5, 21] and desaturases [35, 36]. Irrespective of the mechanism driving the distinct phenotype among Elovl4a enzymes, it is clear that the *C. gariepinus* orthologue was very efficient in the production of VLC-PUFA from exogenously supplemented PUFA substrates. Such elongation capabilities largely apply to the *C. gariepinus* Elovl4b, although a distinctive trait of Elovl4b is its ability to efficiently elongate exogenously added 22:6n-3 to 32:6n-3, a VLC-PUFA that has been detected in retinal phosphatidylcholine of gilthead seabream *Sparus aurata* [37]. Despite the ability of the *C. gariepinus* Elovl4b to produce 32:6n-3 from DHA (22:6n-3), the latter does not appear to be a preferred substrate for biosynthesis of n-3 VLC-PUFA in bovine and rat retina [38, 39]. It was demonstrated that, while exogenously supplemented EPA and 22:5n-3 acted as precursors for VLC-PUFA biosynthesis, DHA was incorporated directly into retinal phospholipids without further metabolism.

Irrespective of whether teleost Elovl4 can utilize DHA or not, their ability to elongate 22:5n-3 to 24:5n-3 suggested that Elovl4 can play a role in DHA biosynthesis through the Sprecher pathway [19]. This pathway requires the production of 24:5n-3 for further Δ6 desaturation and partial β-oxidation to DHA, and Elovl2 has been identified as a major candidate elongase accounting for the provision of 24:5n-3 from 22:5n-3 [4]. *C. gariepinus* possess an Elovl2 with the abovementioned ability to elongate 22:5n-3 to 24:5n-3 [26], indicating that Elovl2 and Elovl4 have partly overlapping functions as previously described between Elovl2 and Elovl5 [5]. In contrast, teleosts within the Acanthopterygii clade have apparently lost Elovl2 [40], and consequently the presence of Elovl4 with the ability to elongate 22:5n-3 is clearly advantageous to compensate for this loss [4]. Studies in mammals have not fully clarified whether ELOVL4 participates in DHA biosynthesis. High expression of *ELOVL4* in tissues where DHA accounted for a large proportion of the PUFA content including retina, brain and testis, along with the crucial role DHA plays in the development and function of these tissues suggested a role of mammalian ELOVL4 in DHA biosynthesis [7, 32, 41, 42]. Moreover, Vasireddy *et al*. [15] reported a reduction in DHA and 22:5n-3 in non-polar lipids and free FA of whole skin of mouse without a functional *ELOVL4* compared to skin from wild type controls. On the contrary, Agbaga *et al*. [7, 16] concluded ELOVL4 did not participate in DHA biosynthesis in mammals or may play a redundant role, the latter hypothesis aligning well with the overlapping roles between Elovl2 and Elovl4 described above.

In conclusion, the present study demonstrated that *C. gariepinus* possess two distinct *elovl4*-like elongases with high homology to the previously described zebrafish Elovl4a and Elovl4b. Both *C. gariepinus* Elovl4 participate in the biosynthesis of both VLC-SFA and VLC-PUFA. While previous studies on teleosts had reported on the ability of Elovl4b-like elongases to operate efficiently towards both saturated and polyunsaturated FA, the herein described ability of the *C. gariepinus* Elovl4a to elongate PUFA was in contrast to that of *D. rerio* Elovl4a, the only Elovl4a-like elongase functionally characterized to date. The tissue distribution of *C. gariepinus elovl4* mRNA largely followed previous observations in other teleosts, with neuronal and reproductive tissues exhibiting the highest expression levels.

## Abbreviations

aa: Amino acid
ABO: Accessory breathing organ
ANOVA: Analysis of variance
ARA: Arachidonic acid
bp: Base pair
cDNA: Complementary DNA
DHA: Docosahexaenoic acid
EFA: Essential fatty acid
Elovl: Elongation of very long-chain fatty acid protein
EPA: Eicosapentaenoic acid
ER: Endoplasmic reticulum
FA: Fatty acid
FAME: Fatty acid methyl ester
Fads: Fatty acyl desaturase
LC-PUFA: Long-chain (≥C_20_) polyunsaturated fatty acid
NTC: No template control
ORF: Open reading frame
PCR: Polymerase chain reaction
PUFA: Polyunsaturated fatty acid
qPCR: Quantitative real-time polymerase chain reaction
SCMM^-ura^: *Saccharomyces cerevisiae* minimal medium minus uracil
VLC-SFA: Very long-chain (>C_24_) saturated fatty acid
VLC-PUFA: Very long-chain (>C_24_) polyunsaturated fatty acid
